# Effects and mechanisms of alveolar type II epithelial cell apoptosis in severe pancreatitis-induced acute lung injury

**DOI:** 10.3892/etm.2013.1453

**Published:** 2013-12-17

**Authors:** GELIANG LIU, JINGWEN ZHANG, HAILONG CHEN, CHAO WANG, YANG QIU, YUEJIAN LIU, JIAJIA WAN, HUISHU GUO

**Affiliations:** 1 Department of General Surgery, First Affiliated Hospital, Dalian Medical University, Dalian, Liaoning 116011, P.R. China; 2 Central Laboratory, Institute of Integrative Medicine, Dalian Medical University, Dalian, Liaoning 116044, P.R. China

**Keywords:** severe pancreatitis, acute lung injury, alveolar type II epithelial cells, Qingyi decoction, intracellular free Ca^2+^

## Abstract

This study aimed to examine the role of alveolar type II epithelial cell (AEC II) apoptosis in severe pancreatitis-induced acute lung injury (ALI) and the intervening role of Qingyi decoction (QYT). An SAP model was established in male Sprague-Dawley rats. Immunohistochemical analysis was conducted to observe the pathological changes in the pancreas and lung tissue. AEC II apoptosis was detected by flow cytometry and the free Ca^2+^ concentration in AECs II was determined by laser scanning confocal microscopy. A radioimmunoassay was performed to determine serum TNF-α content. Quantitative polymerase chain reaction (qPCR) and immunohistochemical analysis were performed to detect the mRNA and protein expression levels of Bax and caspase-8 in the lung tissue. Hematoxylin and eosin staining of lung tissue sections in the severe acute pancreatitis (SAP) group showed pathological changes from control tissue, consistent with acute lung injury (ALI). Flow cytometry showed that the level of AEC II apoptosis in the SAP group was significantly increased compared with that in the control group (P<0.01). Laser scanning confocal microscopy indicated that the free Ca^2+^ concentration in the AECs II of the SAP group was also significantly increased compared with that in the control (P<0.01). Radioimmunoassay demonstrated that the TNF-α levels were significantly increased in the SAP group compared with those in the control group (P<0.01), and qPCR results showed that the levels of Bax and caspase-8 apoptotic gene expression in the AECs II of the SAP group were significantly elevated (P<0.01). The aforementioned indicators were significantly lower following drug treatment compared with the levels observed in the SAP model group. These results suggest that AEC II apoptosis is involved in the ALI procedure associated with SAP. The mitochondrial pathway and death receptor pathway may have key regulatory roles in AEC II apoptosis. The use of QYT may significantly reduce the extent of lung injury.

## Introduction

Patients with severe acute pancreatitis (SAP) commonly present with acute lung injury (ALI) in the early stages; the disease further develops into acute respiratory distress syndrome (ARDS), the earliest concurrent disease with the highest incidence rate amongst patients afflicted with SAP-induced multiple organ system dysfunction (MODS) (1,2). A current focus of SAP treatment is the quest for novel measures for minimizing and preventing lung injury. Clinical studies and animal experiments have shown that the apoptosis of alveolar type II epithelial cells (AECs II) is important in ALI, but that the pathway involved in AEC II apoptosis in severe pancreatitis-induced ALI remains unclear (3–6).

The efficacy of the Chinese medicine, Qingyi decoction (QYT), has been demonstrated by a number of years of clinical practice and animal experiments; QYT is also an effective prescription for the treatment of acute pancreatitis (7). QYT exhibits purgative functions, promotes blood circulation, eliminates blood stasis and reduces inflammation. It directly neutralize endotoxins and also protects the intestinal barrier, reduces intestinal endotoxin generation and absorption, inhibits excessive neutrophil activation, downregulates NF-κB expression and minimizes the release of inflammatory cytokines, including tumor necrosis factor-α (TNF-α) and nitric oxide (NO). Therefore, the bioactivities of QYT include the protection of the lung permeability barrier, prevention of oxidative damage and improvement of the microcirculation (8). In our previous study on the role of QYT in patients following acute pancreatitis, it was shown that QYT administration reduced lung injury by decreasing the expression of secretory type II phospholipase A_2_ at the transcriptional level, thereby protecting pulmonary function (8). Dexamethasone is a glucocorticoid that exhibits significant functions, including anti-inflammatory activity, microcirculation promotion, oxygen free radical scavenging and NF-κB inhibition; it has recently been found to inhibit lung inflammatory responses and excessive lung tissue apoptosis (9). Moreover, QYT combined with dexamethasone has been shown to exert synergistic therapeutic effects on early systemic inflammatory response syndrome associated with SAP (10). Verapamil, a Ca^2+^ channel blocker, reduces the intracellular Ca^2+^ content, and inhibits the production and release of inflammatory cytokines (11). It also exerts protective effects on rat lung injury caused by SAP. The combination of Chinese and Western medicine is currently regarded as an effective approach to curing pancreatitis.

The present study aimed to examine the role of AEC II apoptosis in severe pancreatitis-induced ALI and the intervening role of QYT. QYT, dexamethasone and verapamil were administered as intervening therapies on a rat model of SAP-induced ALI. The results were analyzed in order to clarify the mechanisms affecting AECs in the course of ALI, and may serve as a basis for novel formulations combining traditional Chinese medicine and Western medicine for the therapeutic treatment of SAP-induced ALI.

## Materials and methods

### Animals and grouping

Sixty clean-grade healthy male Sprague-Dawley rats (weight, 180–220 g; age, 8 weeks) were provided by the Experimental Animal Center at Dalian Medical University (Dalian, China). The animals were randomly divided into 5 groups (n=12 per group): The model group (SAP), control group, QYT treatment group, dexamethasone (Zhengzhou Ling Rui Pharmaceutical Co., Ltd. Zhengzhou, China) treatment (DEX) group and verapamil (Shanghai Harvest Pharmaceutical Co., Ltd., Shanghai, China) treatment group (VER). This study was carried out in strict accordance with the recommendations in the EU animal management practices (1986). The animal use protocol was reviewed and approved by the Institutional Animal Care and Use Committee of Dalian Medical University (Dalian, China).

### Model preparation

The SAP group: 10% chloral hydrate aldehyde (4 ml/kg body weight) was intraperitoneally injected as an anesthetic. An ~2-cm long incision was made directly under the xiphoid midline. Once the duodenum was located along the pylorus inside the abdomen, the duodenal papilla opening was exposed, the cholangitic porta hepatis was clipped with a small artery clamp, a 1-ml syringe needle was placed (from the intestinal wall contralaterally to the duodenal nipple) into the biliopancreatic duct through the duodenal papillary opening on the cholo-pancreatic duct and 1.5% sodium deoxycholate (Baier Di Biotechnology Co., Ltd., Beijing, China; 1 ml/kg body weight) was slowly infused retrogradely in 30 sec. Following injection, an appropriate pressure was maintained by hand across the gauze on the biliopancreatic duct through the duodenal papillary for ~3 min, and changes to the pancreatic tissue (e.g. congestion, edema and hemorrhage) were observed. The syringe and arterial clamp were removed and the incision was sutured. Postoperative feeding was conducted in the Research Center of Dalian Medical University, the Central Laboratory of Dalian Central Hospital and the Central Laboratory of First Affiliated Hospital, Dalian Medical University.

The control group: An incision was made in the abdomen of the animals and the pancreatic tissue was marginally rotated several times prior to closing the abdomen.

The treatment groups: The model preparation method was the same as that for the SAP group, but the QYT group was orally treated with QYT (First Affiliated Hospital of Dalian Medical University, Chinese Medicine Preparations Division; 10 ml/kg body weight/dose) 0.5 h prior to model preparation. The DEX group was injected with dexamethasone via the tail vein (2 ml/kg body weight/dose, slow bolus at a speed of 0.3 ml/min), and the VER group was injected with verapamil via the tail vein (0.5 ml/kg body weight/dose, diluted with saline to the volume used in the DEX group, slow bolus at a speed of 0.3 ml/min). The administration was repeated 6 and 12 h postoperatively. The remaining groups received the same volume of saline by intragastric administration. The rats were sacrificed by anesthetic overdose.

### Separation of AECs II

The lung tissues were quickly removed and sliced into 1.0-mm^3^ pieces following lavage. Calf serum was added to the sliced tissues to inactivate trypsin and then phosphate-buffered saline (PBS) solution was added to a total volume of 20 ml. The samples were then placed in a 37°C water bath shaker for 5 min at 24.975 × g. Subsequently, the solution was filtered and the cell filtrate was centrifuged at 352 × g for 10 min. The supernatant was removed and the precipitate was suspended in Dulbecco’s modified Eagle’s medium (DMEM). The cell suspension (10 ml) was placed in a large petri dish pre-coated with rat immunoglobulin G (Beijing Boda Tektronix Biotechnology Co., Ltd., Beijing, China) and incubated for 1 h. The non-adherent cells were gently removed from the centrifuge tube and the suspension was centrifuged at 352 × g for 10 min. The precipitate was resuspended with DMEM containing 10% fetal bovine serum and then the cells were counted.

### Pathological observations

Pathological observations were made under an optical microscope (Leica DMIRB; Leica, Solms, Germany). The lower lobes of the left lung and pancreatic tissue were cut, fixed with 4% paraformaldehyde and dehydrated with gradient alcohol. The lobes were then fixed with xylene and embedded with liquid paraffin to obtain pathological sections for hematoxylin and eosin (H&E) and immunohistochemical staining.

### Detection of apoptosis

Following the instructions provided with the Annexin V-FITC apoptosis detection kit (cat. no. 556547; BD Pharmingen, Franklin Lakes, NJ, USA), AECs II were collected and the concentration was adjusted to 1×10^6^/ml. The cells were incubated with Annexin V-FITC and propidium iodide dye in the dark at room temperature. The Annexin-V binding buffer was then diluted and analyzed with an Epics XL flow cytometer (Beckman Coulter, Miami, FL, USA).

### Detection of Ca^2+^ concentration

To the DMEM-resuspended AEC II suspension was added Ca^2+^ microprobe Fluo-3/AM (500 μl, 10 μmol/l; cat. no. sc-202612; Santa Cruz Biotechnology, Inc., Dallas, TX, USA). The cells were maintained at 37°C in the dark for 30–45 min. The cell suspension was then placed in a dedicated petri dish, from which eight views of each specimen were randomly selected. A laser scanning confocal microscope (Leica TCS SP5) was used for observation of the cells and image capture. The total number of cells and the total fluorescence intensity (FI) per visual field were calculated with Image-Pro Plus software, version 6.0 (Media Cybernetics, Inc., Rockville, MD, USA). The mean FI of individual cells in each visual field was computed and expressed as the intracellular free Ca^2+^ concentration.

### Detection of TNF-α

A radioimmunoassay kit (cat. no. ABIN118027; antibodies-online Inc., Atlanta, GA, USA) was used to determine the serum TNF-α levels according to the manufacturer’s instructions. Results are expressed in ng/ml.

### Quantitative polymerase chain reaction (qPCR)

Total RNA was extracted from AECs II with TRIzol solution (cat. no. 15596–018; Invitrogen Life Technologies, Carlsbad, CA, USA) for reverse transcription. Following reverse transcription, 10 μl cDNA was mixed with the PCR reaction system (primer sequences shown in Table I). The denaturation, annealing and extension temperatures were 94, 56 and 72°C, respectively, and the reaction times were 30, 30 and 1.5 min, respectively. For β-actin and Bax, 30 cycles were conducted and for caspase-8, 35 cycles were conducted. The reaction product (5 μl) was subjected to 2% agarose gel electrophoresis. A gel scanning system [Protein Simple (Alphalmade HP), Santa Clara, CA, USA] was used to scan and analyze the absorbance, provide images and determine the integral optical density (IOD) value of each band, with the results expressed as the IOD ratio of Bax and caspase-8 to β-actin, as relative intensities of Bax and caspase-8 mRNA expression.

### Immunohistochemistry

SP two-step staining was adopted, and the procedure was performed according to the manufacturer’s instructions (Streptavidin-Peroxidase Immunohistochemical staining kit; Histostain-Plus Kit; Zymed Laboratories, San Francisco, CA, USA). Paraffinized lung sections were dewaxed and hydrated, followed by microwave antigen retrieval in citrate buffer, wherein 0.3% H_2_O_2_ was used to remove endogenous peroxidase and 5% sheep serum was used for lutation. The Bax and caspase-8 polyclonal antibodies (cat. no. sc-493 and sc-56070, respectively; Santa Cruz Biotechnology, Inc., Dallas, TX, USA) were added to the sections, which were then incubated at 4°C overnight. After washing with PBS, the sample was incubated with biotin-labeled secondary antibody (cat. no. SAB3700835; Sigma-Aldrich, St. Louis, MO, USA) for 1 h at room temperature, then stained with 3,3′-diaminobenzidine, re-stained with hematoxylin, dehydrated, hyalinized and mounted. Dark staining detected by light microscopy indicated a positive reaction and PBS was used in the negative control group. Staining intensity was determined with optical density (OD) values, and the concentration of Bax and caspase-8 proteins were expressed as the mean OD (total IOD/total area).

### Statistical analysis

All data were statistically analyzed with SPSS software, version 11.5 (SPSS, Inc., Chicago, IL, USA). The experimental values are expressed as the mean ± standard deviation. The differences between the treatment group and the control group were compared via Student’s t-test. P<0.05 was considered to indicate a statistically significant difference. The correlation between indicators was analyzed by Pearson product-moment correlation analysis.

## Results

### Pathological changes

**Figure 1:**
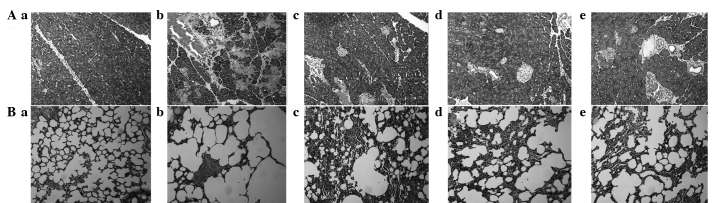
Pathological changes to the pancreas and lung tissues of different experimental model groups. Pathological sections of (A) pancreatic tissue and (B) lung tissues (magnification, ×10). (a) Control group; (b) SAP group; (c) QYT group; (d) DEX group; and (e) VER group. SAP, severe acute pancreatitis; QYT, Qingyi decoction; DEX, dexamethasone; VER, verapamil.

To detect the effects of QYT, dexamethasone and verapamil on the experimental animal models, the pathological changes in the pancreas and lung tissues of different experimental groups were observed with an optical microscope. The control group showed normal pancreas (Fig. 1Aa) and lung tissue morphology (Fig. 1Ba), whereas the SAP group exhibited pancreatic changes indicated by acinar destruction, interstitial congestion, edema and inflammatory cell infiltration (Fig. 1Ab). The changes in the lung tissues corresponded with ALI changes, appearing as marked pulmonary edema and alveolar septum damage, as well as a small concentration of white blood cells and a large number of red blood cells in the alveolar cavity with the retention and aggregation of neutrophils (Fig. 1Bb). The DEX (Fig. 1Ad and Bd) and VER groups (Fig. 1Ae and 1Be) showed significant improvements compared with the SAP group, but pulmonary edema and neutrophil infiltration remained. The effectiveness of treatment in the QYT group (Fig. 1Ac and 1Bc) was lower than that of the SAP group.

### AEC II apoptosis

**Figure 2:**
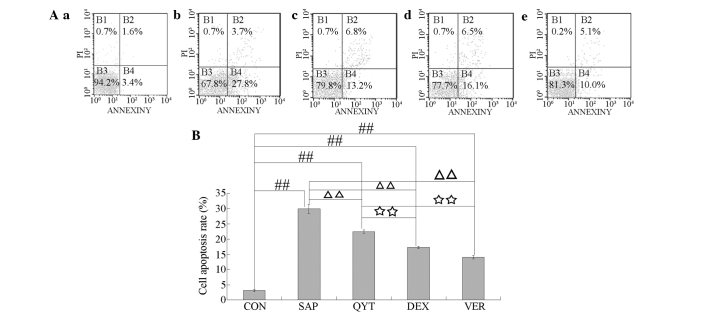
(A) AEC II apoptosis flow cytometry results in the (a) control group; (b) SAP group; (c) QYT group; (d) DEX group; and (e) VER group. (B) AEC II apoptosis rates in the various groups. AEC II, alveolar type II epithelial cells; SAP, severe acute pancreatitis; QYT, Qingyi decoction; DEX, dexamethasone; VER, verapamil. ^##^P<0.01 vs. CON group; ^ΔΔ^P<0.01 vs. SAP group; ^⋆⋆^P<0.01 vs. DEX group.

The AEC II apoptosis rates of the different experimental groups were analyzed by flow cytometry, and the results are shown in Fig. 2. Compared with the AEC II apoptosis rate of the control group (Fig. 2Aa), those of the SAP, QYT, DEX and VER groups (Fig. 2Ab–e) were significantly increased (P<0.01). The AEC II apoptosis rates of the three treatment groups were significantly decreased compared with that of the SAP group (P<0.01 Fig. 2B). The AEC II apoptosis rates of the DEX and VER groups were significantly decreased compared with that of the QYT group (P<0.01; Fig. 2B), and the AEC II apoptosis rate of the VER group was significantly decreased compared with that of the DEX group (P<0.01; Fig. 2B).

### Intracellular free Ca^2+^ concentration

**Figure 3:**
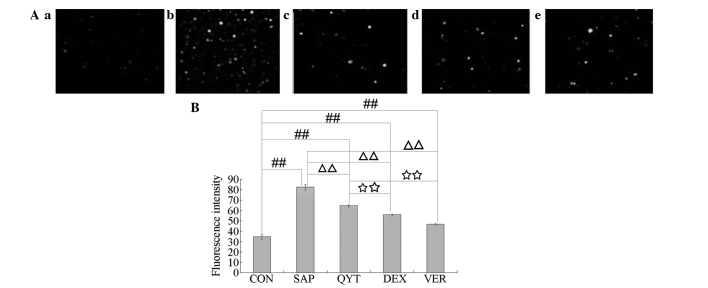
(A) Free Ca^2+^ concentration fluorescence map of AEC II in the (a) control group; (b) SAP group; (c) QYT group; (d) DEX group; and (e) VER group. (B) Intracellular free Ca^2+^ concentration as evaluated by fluorescence intensity. AEC II, alveolar type II epithelial cells; SAP, severe acute pancreatitis; QYT, Qingyi decoction; DEX, dexamethasone; VER, verapamil. ^##^P<0.01 vs. CON group; ^ΔΔ^P<0.01 vs. SAP group; ^⋆⋆^P<0.01 vs. VER group.

To investigate the role of Ca^2+^ in AEC II apoptosis in SAP-induced lung injury, laser scanning confocal microscopy was conducted to measure the intracellular free Ca^2+^ concentration. The results are shown in Fig. 3. Compared with the intracellular FI of the control group (Fig. 3Aa and 3B), those of the SAP group (Fig. 3Ab and 3B) and the QYT, DEX and VER treatment groups (Fig. 3Ac–e and 3B) were significantly increased (P<0.01). The intracellular FIs of the QYT, DEX and VER treatment groups were significantly decreased compared with that of the SAP group (P<0.01). The intracellular FIs of the DEX and VER groups were significantly decreased compared with that of the QYT group (P<0.01), and the intracellular FI of the VER group was significantly decreased compared with that of the DEX group (P<0.01).

### Serum TNF-α content and correlation analysis

To elucidate the role of TNF-α and other inflammatory cytokines in AEC II apoptosis caused by SAP-induced lung injury, a radioimmunoassay was used to measure the serum TNF-α levels. Table II shows that compared with the TNF-α levels of the control group, those of the SAP and the DEX, QYT and VER treatment groups were significantly increased (P<0.01). The TNF-α levels of the three treatment groups were significantly decreased compared that of the SAP group (P<0.01). The TNF-α levels of the DEX and VER groups were significantly decreased compared with that of the QYT group (P<0.01), and the TNF-α level of the VER group was significantly decreased compared with that of the DEX group (P<0.01). Correlation analysis of the AEC II apoptosis index, intracellular free Ca^2+^ concentration and serum TNF-α content indicated that the apoptotic index was positively correlated with the intracellular free Ca^2+^ concentration and serum TNF-α content (Table III).

### Bax and caspase-8 mRNA expression and correlation analysis

**Figure 4:**
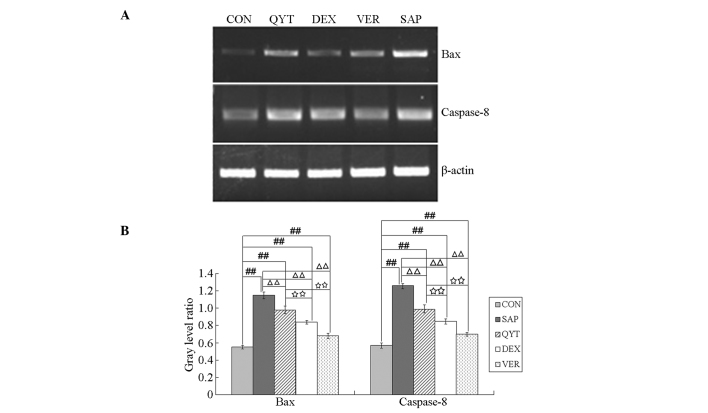
(A) qPCR product electrophoresis chromatographs of Bax, caspase-8 and β-actin mRNA. (B) Gray value ratio comparison of Bax and caspase-8 to β-actin. qPCR, quantitative polymerase chain reaction; CON, control; SAP, severe acute pancreatitis; QYT, Qingyi decoction; DEX, dexamethasone; VER, verapamil. ^##^P<0.01 vs. CON group; ^ΔΔ^P<0.01 vs. SAP group; ^⋆⋆^P<0.01 vs. VER group.

To clarify the roles of Bax, caspase-8 and other apoptosis-associated proteins in AEC II apoptosis caused by SAP-induced lung injury, the expression levels of Bax and caspase-8 mRNA in the AECs II of the different experimental groups were analyzed. The results are shown in Fig. 4. The gray level ratios for Bax and caspase-8 mRNA expression in the control group were significantly lower than those in the SAP, QYT, DEX and VER groups (P<0.01). The gray level ratios in the SAP group were significantly higher than those of the QYT, DEX and VER groups (P<0.01). The gray level ratios in the QYT group were significantly higher than those in the DEX and VER groups (P<0.01), and the gray level ratios in the DEX group were significantly higher than those in the VER group (P<0.01). Correlation analysis of the ACE II apoptosis index and the expression levels of Bax and caspase-8 mRNA indicated that the apoptotic index was positively correlated with the expression levels of Bax and caspase-8 mRNA (Table IV).

### Expression of Bax and caspase-8

**Figure 5:**
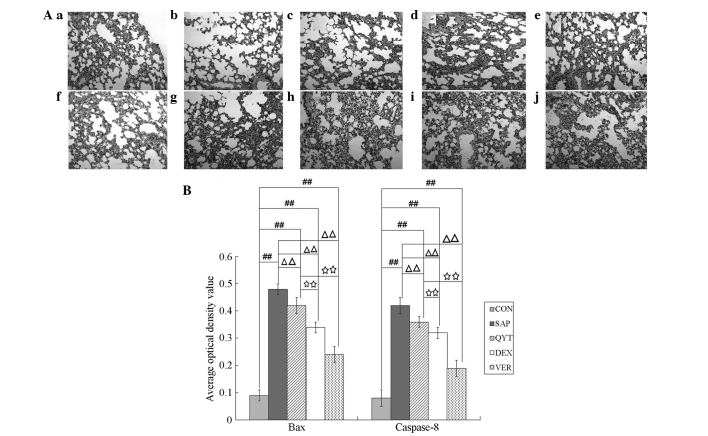
(A) Immunohistochemical analysis was used to detect Bax and caspase-8 protein expression in the lung tissues of different experimental groups. (B) Mean optical density comparison of Bax and caspase-8 protein expression. Bax protein expression in the (a) control group; (b) SAP group; (c) QYT group; (d) DEX group; and (e) VER group. Caspase-8 protein expression in the (f) control group; (g) SAP group; (h) QYT group; (i) DEX group; and (j) VER group. (Magnification, ×10). CON, control; SAP, severe acute pancreatitis; QYT, Qingyi decoction; DEX, dexamethasone; VER, verapamil. ^##^P<0.01 vs. CON group; ^ΔΔ^P<0.01 vs. SAP group; ^⋆⋆^P<0.01 vs. VER group.

Immunohistochemical analysis was performed to detect the expression of Bax and caspase-8 proteins in the lung cells of the different experimental groups. The results demonstrated that the mean OD values of Bax and caspase-8 protein expression in the control group (Fig. 5a and f) were significantly lower than those in the SAP (Fig. 5b and g), QYT (Fig. 5c and h), DEX (Fig. 5d and i) and VER (Fig. 5e and j) groups (P<0.01). The mean OD values of Bax and caspase-8 protein expression in the SAP group were significantly higher than those in the QYT, DEX and VER groups (P<0.01). The mean OD values of Bax and caspase-8 protein expression in the QYT (Fig. 5c and h) group were significantly higher than those in the DEX (Fig. 5d and i) and VER (Fig. 5e and j) groups (P<0.01). The mean OD value of caspase-8 protein expression in the DEX (Fig. 5i) group showed no significant difference (P>0.05), whereas that in the VER group (Fig. 5j) exhibited a significant difference (P<0.01). The mean OD value of Bax and casepase-8 protein expression in the VER group (Fig. 5e and j) was significantly lower than that of the DEX group (Fig. 5d and i) (P<0.01).

## Discussion

SAP-induced ALI is a component of systemic inflammatory response syndrome and systemic anti-inflammatory response syndrome in acute pancreatitis. It is a complication in MODS, which is associated with SAP occurring at the earliest stage and associated with the highest mortality rate (1,2)

The ALI/ARDS-induced explosive inflammatory response of the alveolar wall is closely associated with neutrophils (12), alveolar macrophages (13), airway epithelial cells (14) and pulmonary vascular endothelial cells (15). These cells produce numerous inflammatory factors that are closely associated with apoptosis.

Recent studies have shown that AECs, particularly AECs II, as progenitor cells play a critical role in apoptosis in the pathological process of ALI (16). In the study of apoptosis-regulating genes, the bcl-2 family, Fas-FasL and the caspase family are commonly investigated (17). Bcl-2/Bax is embedded in the mitochondrial membrane, and regulates the release of apoptosis-inducing proteins and mitochondrial functions (18). Unphosphorylated Bcl-2-associated death promoter (Bad) directly causes the haplomerization of Bax, thereby forming the mitochondrial permeable exchange channel, which results in mitochondrial Ca^2+^ influx and cytochrome *c* outflow. These phenomena reduce membrane potential, result in insufficient ATP formation and caspase activation, activate a series of downstream apoptotic genes and induce apoptosis. The present study aimed to identify the role of AEC II apoptosis in severe pancreatitis-induced ALI, as well as the intervening role of QYT. Bax expression was observed to be minimal in normal lung tissues, significantly enhanced in ALI lung tissues and positively correlated with the alveolar cell apoptosis index, consistent with previous studies (19,20). Caspase family members are the key effector molecules of apoptotic signal transduction and molecular performers of apoptosis; their activation is the key biochemical event for apoptotic effects (5). As a crucial caspase, caspase-8 activates caspase-3, as well as stimulating its hydrolysis and activating polymerase, thereby inducing cell death (21). The results suggest that, as the key promoter in the Fas-FasL-mediated apoptotic pathway and mitochondrial-mediated apoptotic pathway, caspase-8 mRNA and protein expression levels significantly increase with increasing AEC apoptosis in ALI.

Controlling the intensity of inflammation in SAP is essential for the body to maintain a steady state. Recent studies have shown that glucocorticoids exert protective effects against lipopolysaccharide-induced rat ALI, which may be associated with inhibition of the expression of inflammatory cytokines, such as TNF-α and IL-1β (22). Dexamethasone has been shown to inhibit Fas antibody and interferon-γ, and mitigate AEC apoptosis. A previous study indicated that methylprednisolone is able to inhibit the pulmonary inflammatory response and excessive apoptosis of lung tissues, confirming that glucocorticoids regulate lung tissue apoptosis (23). As a glucocorticoid, dexamethasone has significant functions, such as anti-inflammatory, microcirculation-improving, oxygen free radical-scavenging and NF-κB-inhibiting roles. The present study demonstrated that dexamethasone significantly decreased the TNF-α and serum amylase levels compared with those in rats with SAP. Pancreatic and lung injury significantly decreased and the AEC II apoptosis rate significantly decreased in the dexamethasone-treated rats. Moreover, the expression of apoptotic Bax and caspase-8 at the mRNA and proteins levels significantly decreased. Even though dexamethasone is a strong immunosuppressant, long-term and substantial administration causes severe body immunosuppression, thereby increasing infection and other complications, as well as mortality due to sepsis. These effects have resulted in considerable controversy with regard to its application. However, from the perspective of the effects on SAP lung injury, dexamethasone is able to inhibit inflammation, stabilize lysosomal enzymes, promote the secretion of pulmonary surfactants and protect AECs, exerting a certain therapeutic effect in ARDS patients. However, the timing of application, dosage and course of treatment require further exploration.

Calcium channel blockers have been shown to exert protective effects against SAP-induced lung injury and such effects may be associated with the following factors: i) Verapamil, a calcium channel blocker, inhibits the increase in intracellular Ca^2+^, thereby preventing the production and release of inflammatory cytokines (24,25). ii) Verapamil also inhibits exocrine secretion by the pancreas and pancreatic enzyme activities; iii) it may reduce thromboxane content, stabilize the ratio of thromboxane/prostaglandin I (26), inhibit platelet aggregation and attenuate the microcirculation disturbance of the pancreas, thereby reducing pathological lesions in the pancreas. iv) Verapamil may reduce intracellular calcium overload and reduce calcium overload-induced apoptosis (27,28). The results of the present study suggest that compared with the serum TNF-α levels and serum amylase of the SAP group, those of the VER group significantly decreased. Lung and pancreas damage, AEC II apoptosis and the expression of Bax and caspase-8 mRNA also significantly decreased. The 5 h postoperative situation of the VER group was not as good as that of the DEX group after modeling 5 h, a result that may be associated with the side-effects of calcium channel agents. The appropriate application and dosage require further study.

Approximately 1,700 years ago, Zhang Zhongjing stated in ‘Medical Treasures of the Golden Chamber’ that pressing ‘would make the heart full of pain and the sudden death should be applied Da Chaihu Tang’. This phrase describes conditions that are highly similar to the main symptoms of acute pancreatitis. The Chinese prescription, QYT, is effective against acute pancreatitis as indicated by years of clinical and experimental (animal) application. It may exhibit purgative functions, promote blood circulation, eliminate blood stasis and reduces inflammation. It not only directly neutralizes endotoxins, but also protects the intestinal barrier, reduces the generation and absorption of intestinal endotoxins, inhibits the excessive activation of neutrophils, downregulates NF-κB expression and minimizes the release of inflammatory cytokines, such as TNF-α and NO. Therefore, its bioactivities protect the lung permeability barrier, prevent oxidative damage and improve microcirculation. The results of the present study showed that using QYT significantly improved the symptoms of intestinal obstruction in SAP rats. Compared with the serum TNF-α and amylase levels of the SAP group, those of the remaining treatment groups significantly decreased. Lung and pancreatic damage, AEC II apoptosis and the expression levels of Bax and caspase-8 mRNA also significantly decreased (29).

In conclusion, the results of the present study demonstrated that AEC II apoptosis participates in SAP-induced ALI, and that the mitochondrial pathway and death receptor pathway have regulatory roles in AEC II apoptosis. The application of QYT, dexamethasone and verapamil significantly reduced the extent of lung injury. These results suggest that QYT is beneficial for the treatment of SAP-induced ALI. Further studies are required to investigate whether a synergistic effect occurs when traditional Chinese medicine and Western medicines are combined.

## Figures and Tables

**Table I:** Primers and product sizes of polymerase chain reaction.

Product	5′-Primer	3′-Primer	Size (bp)
β-actin	TCATGAAGTGTGTTGACATCCGTAAAG	CCTAGAAGCATTTGCGGTGCACGATGGACG	285
Bax	CTGAGCTGACCTTGGAGC	GACTCCAGCCACAAAGATG	413
Caspase-8	TGATGAAGAGGCTCTGAGTAA	TGGCAAAGTGACTGGATATA	489

**Table II:** Serum TNF-α levels of different groups.

Group (n=12)	TNF-α (ng/ml)
Control	0.93±0.47
SAP	5.09±0.12^a^
QYT	3.95±0.92^a^,^b^
DEX	3.41±0.11^a^-^c^
VER	2.04±0.72^a^-^d^

a P<0.01 compared with the control group;

b P<0.01 compared with the SAP group;

c P<0.01 compared with the QYT group; and

d P<0.01 compared with the DEX group.

TNF-α, tumor necrosis factor-α; SAP, severe acute pancreatitis; QYT, Qingyi decoction; DEX, dexamethasone; VER, verapamil.

**Table III:** Correlation analysis of AEC II apoptosis index with intracellular free Ca^2+^ concentration and serum TNF-α level.

Statistic	Ca^2+^	TNF-α
R-value	0.984	0.987
P-value	<0.01	<0.01

AEC II, alveolar type II epithelial cell; TNF-α, tumor necrosis factor-α.

**Table IV:** Correlation analysis of AEC II apoptosis index with the expression levels of Bax and caspase-8 mRNA.

Statistic	Bax	Caspase-8
R-value	0.979	0.97
P-value	<0.01	<0.01

AEC II, alveolar type II epithelial cell.
